# Benefits of Social Contact in Individuals With Psychotic Symptoms: Do Closeness of the Contact and Empathic Skills Make the Difference?

**DOI:** 10.3389/fpsyg.2021.769091

**Published:** 2021-12-16

**Authors:** Lisa J. G. Krijnen, Imke L. J. Lemmers-Jansen, Anne-Kathrin J. Fett, Lydia Krabbendam

**Affiliations:** ^1^Child and Adolescent Studies, Utrecht University, Utrecht, Netherlands; ^2^Department of Clinical, Neuro and Developmental Psychology, Faculty of Behavioural and Movement Sciences, Vrije Universiteit Amsterdam, Amsterdam, Netherlands; ^3^Institute for Brain and Behavior (IBBA), Amsterdam, Netherlands; ^4^CSI Lab, Department of Psychosis Studies, Institute of Psychiatry, Psychology and Neuroscience, London, United Kingdom; ^5^Department of Psychology, City University of London, London, United Kingdom

**Keywords:** first episode psychosis (FEP), clinical high risk (CHR) for psychosis, social contact, close contact, positive psychotic symptoms, positive and negative affect, experience sampling method (ESM)

## Abstract

**Objectives:** Social contact is known to be beneficial for humans’ mental health. Individuals with psychotic symptoms (PS) tend to show poorer social and interpersonal functioning. However, in this patient population, social contact may be crucial for their mental wellbeing and treatment success. Additionally, closeness of social contact (familiar versus less familiar others), rather than only the presence or absence of social contacts, may play an important role. Empathy may heighten the beneficial effects of social/close contact on mental health, facilitating interactions. We investigated the association between social contact and closeness of contact on mental health, defined as positive symptoms, positive affect and negative affect in PS and control participants, with empathy as a moderator.

**Methods:** Participants were 16–30 years old. Information regarding social/close contact and mental health was obtained using the experience sampling method in individuals with PS (*n* = 29) and healthy controls (*n* = 28). Empathy was measured using a self-report questionnaire.

**Results:** Social contact was associated with higher positive affect in the total sample. Contact with close as opposed to less close others was related to better mental health: It was associated with lower positive symptoms in the PS group, and with more positive affect in the total sample. Empathy moderated the association between closeness of contact and positive affect in the total sample, in which the combination of higher levels of empathy combined with the presence of close contact was associated with higher positive affect in the total sample. However, the direct association between empathy and positive affect was not significant per group of contact.

**Conclusion:** The results suggest that social contact, but especially contact with a close other is important for mental health outcomes: Contact with close others is beneficial for positive affect in the total sample and for positive symptoms in individuals with PS.

## Introduction

Psychotic disorders, including schizophrenia are severe conditions that have a significant impact on daily functioning of individuals experiencing psychotic symptoms (PS) (Cho et al., 2017). These disorders are characterized by positive symptoms (e.g., delusions and hallucinations), negative symptoms, such as diminished emotional expression and avolition, disorganized thinking (speech), grossly disorganized or abnormal motor behavior, and cognitive dysfunctions (American Psychiatric Association [APA], 2013). Psychotic disorders are also associated with poorer social and interpersonal functioning (Yager and Ehmann, 2006). However, a possible protective factor against the symptoms in this patient group is social contact. For individuals with PS, social contact has been linked to mental health and psychological wellbeing (Bengtsson-Tops and Hansson, 2001; Meyer-Lindenberg and Tost, 2012; Bjornestad et al., 2017). The concern, however, is that individuals with PS, including people at clinical high risk (CHR) for developing psychosis, have significantly poorer social networks in terms of quantity and quality of contact than healthy individuals (Bengtsson-Tops and Hansson, 2001; Pruessner et al., 2011), though social contact might be particularly important for this patient group.

Impaired social functioning (e.g., in terms of the number of close friends, quality of the friendship, number of social contacts, etc.) has been found to be predictive for developing a psychotic disorder in individuals at CHR (Cornblatt et al., 2007). In patients with schizophrenia, it was shown that a lack of social contact was predictive for more severe negative symptoms, lower psychosocial functioning and a worse quality of life (Millier et al., 2014). These findings support the idea that social contact is related to better outcomes for individuals with, or at high risk for, psychotic disorders.

The closeness of the contact might be the crucial element that determines whether social contact is beneficial or not. Research showed that though patients with psychotic disorders experienced more paranoia when being alone compared to having social company, both healthy people and patients experienced more paranoia when meeting with distant others compared to close others and that meeting close others was associated with lower paranoia over time (Fett et al., 2021). In a non-clinical sample of individuals at increased risk for psychosis, meeting with less close individuals was found to be associated with an increased risk of experiencing unusual perceptions when compared to being alone, whereas the presence of close contacts was associated with a lower risk (Verdoux et al., 2003). Individuals with low and medium paranoid traits showed more paranoia when being in company of less close individuals, than in company of close individuals (Collip et al., 2011). However, this association was not significant in individuals with high paranoia. In addition, it was found that individuals who were prone to psychosis showed an increase in negative affect (i.e., anxious and depressed moods) when they were likely to meet with less close others (Husky et al., 2004). These studies support the idea that the beneficial effect of social contact on mental health depends on the closeness of the contact.

The positive effect of social/close contact on mental health might be driven by empathy. Baron-Cohen and Wheelwright (2004) stated that “empathy is the glue of the social world” (p. 163), and it has been defined as the ability to understand and experience the thoughts and feelings of others. Generally, the ability to *understand* one’s thoughts and feelings is called cognitive empathy, whereas the extent to being able to *experience* others’ thoughts and feelings is referred to as affective empathy (Hogan, 1969). Empathy seems to be one of humans’ core abilities to interpret actions of others (Price and Archbold, 1997), which is highly relevant when being in company of others. Empathy has been linked to prosocial and positive behaviors toward others, facilitating interactions and relationships (McDonald and Messinger, 2011). If empathy is lacking, the essence of a social exchange may get lost as the person would not fully understand or “feel” emotions of the other person. This can result in less satisfactory contact which in turn may affect mental health. Therefore, levels of empathy may determine the extent to which a person benefits from social contact in terms of their mental health. In other words, higher levels of empathy could boost the advantages of social contact on mental health.

Although the relationship between empathy, social/close contact and mental health is not clear yet, both empathy and social functioning show abnormal patterns in individuals with PS (Yager and Ehmann, 2006; Montag et al., 2007) and seem to be predictive for functional outcome (e.g., social skills and community functioning) (Brüne, 2005; Bora et al., 2006; Fett et al., 2011). These individuals tend to withdraw from social contact, which in turn negatively influences their social relationships. Abnormalities in social functioning and empathy are found along the psychosis continuum, in patients that experienced multiple psychotic episodes (Grant et al., 2001; Mazza et al., 2012), in first-episode psychosis patients (FEP) (Priebe et al., 2000; Grant et al., 2001; Mazza et al., 2012), and also in CHR (Häfner et al., 1999; Yung et al., 2004; Van Donkersgoed et al., 2015). Previous studies have investigated levels of empathy in these groups. In schizophrenia patients, cognitive empathy was found to be impaired (Montag et al., 2007, 2020; Derntl et al., 2009; Corbera et al., 2021), but results regarding affective empathy were inconsistent with some studies reporting impairments in affective empathy (Derntl et al., 2009; Corbera et al., 2021), and others finding no impairment in both behavior measures and self-rated affective empathy compared to healthy controls (Montag et al., 2007; Berger et al., 2019). Furthermore, affective empathy has been found to be impaired in CHR compared to both schizophrenia patients and healthy controls, but cognitive empathy remained intact in this CHR group (Montag et al., 2020). Since social/close contact is associated with mental health and symptom severity, and empathy might strengthen this relationship, it is important to investigate the link between these factors in individuals with FEP and at CHR.

The aim of the current study was to investigate the link between being alone versus being in social contact, the closeness of the contact (with close versus less close others), empathy and mental health in individuals with PS, compared to healthy controls. Mental health consisted of three constructs: positive affect, negative affect and positive symptoms. Two research questions were addressed: (1) Is social contact compared to being alone associated with differential mental health (i.e., positive symptoms and positive/negative affect) in controls versus individuals with PS, and does the closeness of the contact (close versus less close others) affect mental health differently? (2) Does empathy function as a moderator in these associations? Based on the existing literature, we could not make a prediction regarding the direction of the effect of social contact on mental health, since the nature of the social contact (i.e., close versus less close) seems to play a pivotal role in this relationship. We hypothesized that close contact has a more beneficial effect on mental health than less close contact based on studies linking close contact to higher perceived social support and better outcomes (Nangle et al., 2003; Verdoux et al., 2003; Husky et al., 2004; Collip et al., 2011; Kingery et al., 2011; Manago et al., 2012). We expected this relation to be similar in individuals with PS and healthy participants (Verdoux et al., 2003; Collip et al., 2011). For the second research question, it was hypothesized that empathy functions as a moderator or a “booster,” meaning that participants with high levels of empathy will benefit more from social contact as compared to being alone than participants with lower levels of empathy, as can be seen in lower levels of negative affect and positive symptoms, and more positive affect. It will also be explored whether empathy influences the relationship between closeness of the contact and mental health in individuals with PS and healthy participants.

## Materials and Methods

### Participants

The current study was an add on to a larger study (Lemmers-Jansen et al., 2018, 2019) and included 37 individuals with PS and 30 healthy individuals. However, 8 individuals with PS and 2 controls were excluded because they had less than 20 experience sampling method (ESM) measurements. The final sample therefore consisted of 29 individuals with PS and 28 controls. CHR and FEP participants together formed the PS group, as studies have shown that both patient groups suffer from similar symptoms related to comparable impairments in social functioning (Niendam et al., 2006; Yung et al., 2008; Fusar-Poli et al., 2010; Pruessner et al., 2011).

Individuals of the PS group were recruited in the Academic Medical Center Amsterdam (AMC), the Amsterdam early intervention team psychosis (‘Vroege Interventie Psychose’ – VIP team) and PsyQ in The Hague and were contacted by their treating clinicians. Healthy controls were recruited at vocational and higher educational institutes in the area of Amsterdam and The Hague and were matched on education level, age, and sex. FEP patients were diagnosed at the AMC hospital by using DSM-IV criteria (American Psychiatric Association [APA], 2004), and included in the study within 18 months after diagnosis. CHR individuals were referred to PsyQ by their general practitioner or by other health care professionals. All new admissions were screened using the Comprehensive Assessment of At-Risk Mental States [CAARMS; (Yung et al., 2005)] which is a semi-structured interview used to assess psychotic experiences in the last year. Furthermore, patients had to obtain a score below 55 on the Social and Occupational Functioning Assessment Scale [SOFAS (Goldman et al., 1992; Morosini et al., 2000)] which indicates problems in work/study, relationships and self-care. CHR individuals were included in the study <1 year after the CAARMS assessment. In FEP and CHR, severity of symptoms was assessed with the Positive and Negative Syndrome Scale [PANSS; (Kay et al., 1987)]. Scores did not differ between CHR and FEP on the PANSS subscales and the total score, supporting the decision to merge the CHR and FEP participants into one patient group (PANSS total mean score CHR = 1.96 and FEP = 1.97, *p* = 0.96). The total symptom score of 58.52 of the patient group refers to “mildly ill” (Leucht et al., 2005). See Table 1 for the participants’ characteristics.

**TABLE 1:** Participant characteristics.

Variable	Control group (*n* = 28)	PS group^†^ (*n* = 29)
**Sex**		
Male (%)	15 (53.57%)	13 (44.83%)
**Age**		
Mean (*SD*)	20.36 (2.73)	21.33 (2.91)
Range	16–26	17–30
**Education level^‡^**		
Low, *n* (%)	14 (50.00%)	16 (57.14%)
Medium, *n* (%)	7 (25.00%)	7 (25.00%)
High, *n* (%)	7 (25.00%)	5 (17.86%)
**Country of birth**		
Netherlands, *n* (%)	25 (89.29%)	22 (78.57%)
**Empathy**		
*Mean* (*SD*)^§^	19.93 (3.22)	19.79 (3.17)
**Medication type**		
Antipsychotics, *n* (%)	–	10 (35.71%)
Other medication, *n* (%)^¶^	–	9 (32.14%)
No medication, *n* (%)	–	9 (31.03%)
**PANSS scores^a^**		
Positive symptoms, *M item* (*SD*)	–	1.77 (0.67)
Negative symptoms, *M item* (*SD*)	–	2.23 (0.71)
General symptoms, *M item* (*SD*)	–	1.91 (0.51)
Total mean score, *M (SD)*	–	1.97 (0.49)
Total sum score, *M (SD)*	–	58.52 (14.29)

*SD, standard deviation; M, mean; PANSS, positive and negative syndrome scale; PS group, individuals with psychotic symptoms.*

*^†^The patient group consists of both FEP and CHR.*

*^‡^Low, lower (pre-)vocational education (VMBO, MAVO, and MBO); Medium, higher (pre-)vocational education (HAVO and HBO); High, (pre-) university education (VWO and WO). One missing value in PS group. ^§^One missing value of empathy in the control group and one missing value in the PS group.*

*^§^One missing value of empathy in the control group and one missing value in the PS group. Subscales for affective and cognitive empathy did not differ between groups.*

*^¶^Other medication consisted of antidepressants, benzodiazepines and in one case anticonvulsant medication. Medication use was unknown for one participant.*

*^a^PANSS data were only available for the PS group. Data of four participants were missing.*

Sufficient knowledge of the Dutch language was required. An exclusion criterion for all participants was an IQ < 80, approximately. Individuals with PS were excluded when diagnosed with a primary diagnosis of a mood disorder or comorbid autism spectrum disorder (ASD). Healthy controls were excluded if they had a (family) history of psychiatric disorders or ASD.

### Materials

#### Experience Sampling Method

The ESM, also referred to as Ecological Momentary Assessment (Stone and Shiffman, 1994), was developed to measure behavior, experiences and environment of daily life in a systematic and valid way (De Vries, 1992; Csikszentmihalyi and Larson, 2014). In the current study, the questions of the ESM were delivered on an iPod that the participants were carrying. Questionnaires were sent 10 times a day, for seven continuous days between 7.30 am and 22.30 pm. Within time frames of 1.5 h, measurements came at random intervals, but at least 15 min apart. Participants were alerted by a beep and had 15 min to fill out the questionnaire. The initial questionnaire consisted of 50 items, of which we used 17 items as these were the items measuring social/close contact and mental health (i.e., positive and negative affect and positive symptoms) and we used one additional item regarding to what extent the participant felt comfortable in the current company (see Appendix A).

##### Social Contact

The question “With whom am I now?” was used to investigate whether the person was in social contact. If the person answered that s/he was not with other people, the answer was coded as “alone.” If the person answered that she/he was with others (answer options: “classmates,” “colleagues,” “friends,” “1 friend,” “partner,” “family,” “housemates,” and “stranger”), it was coded as being in social contact.

##### Closeness of Contact

To investigate the closeness of contact, the same item was used as for measuring social contact. However, only answers referring to being with others were taken into account, as being alone did not form part of this concept. A distinction was made between close and less close contacts. In order to do so, we followed the procedure of Fett et al. (2021). Additionally we used the ESM question in which participants were asked to rate on a 7-point Likert scale whether they felt comfortable in the current company (1 = totally disagree to 7 = totally agree) (see Appendix B for more information). Based on this second method, results showed that housemates better fitted the concept of a close contact whereas Fett and colleagues grouped them as less close contacts. Therefore, the final distinction was as follows: Close contacts were considered “friends,” “1 friend,” “partner,” “housemates,” and “family,” whereas “classmates,” “colleagues,” and “stranger” were considered as less close contacts. The contacts that were categorized as close contacts received significantly higher scores on the item regarding feeling comfortable within the company (*p* < 0.001) than the contacts that were considered as less close contacts.

##### Mental Health

Mental health was measured by 16 items, assessing positive symptoms (8 items, e.g., “I hear voices”), positive affect (4 items, e.g., “I feel relaxed”) and negative affect (4 items, e.g., “I feel down”) (Myin-Germeys et al., 2005). We calculated the internal consistency, reflecting an acceptable to good reliability (positive symptoms; α = 0.88, positive affect, α = 0.71, negative affect α = 0.83). Answer options ranged from 1 = “totally disagree” to 7 “totally agree.” Mean scores per outcome category were calculated, with higher scores reflecting more severe positive symptoms, higher negative affect, and higher positive affect. For an overview of the included questions, see Appendix A.

#### Positive and Negative Syndrome Scale

The PANSS is a semi-structured interview which aims to investigate the severity of the psychotic symptoms over the last 2 weeks (Kay et al., 1987). In the current study, the PANSS was used to investigate patients’ symptoms at baseline. The scale consists of three subscales, measuring positive symptoms (7 items), negative symptoms (7 items) and general psychopathology (16 items). All items are rated on a 7-point Likert scale. Mean scores per subscale and sum scores for the total scale were calculated, with a higher score reflecting more severe symptoms. Total scores of 58, 75, 95, and 116 refer to “mildly ill,” “moderately ill,” “markedly ill,” and “severely ill,” respectively (Leucht et al., 2005).

#### Empathy Questionnaire for Children and Adolescents

Empathy was measured by using the EmQue-CA (Overgaauw et al., 2017). This is a 14-item self-report questionnaire measuring one’s empathy during the past 2 months, consisting of three subscales. For the current study, only two subscales have been used: (1) Affective empathy, consisting of 6 items measuring the emotional arousal one experiences when confronted with other’s emotion, and (2) Cognitive empathy, consisting of 3 items measuring the extent to which one understands other’s emotions. A three-point scale was used, with 1 = “not true,” 2 = “somewhat true,” to 3 = “true.” The sum score of these 9 items reflected the level of empathy, with a higher score indicating higher levels of empathy. The EmQue-CA has shown to be a reliable and valid instrument (Overgaauw et al., 2017).

### Procedure

Participants were told that this study focused on social decision making in adolescents/young adults with and without psychosis/abnormal experiences. If the participant was willing to participate, one of the researchers made a home visit to provide the participant with the iPod after the informed consent form was signed. The participant filled in the questionnaire once together with the researcher, to ensure comprehension of the questions and the device. Under the age of 18, one of the parents also signed the informed consent. A week later the iPod was returned and the larger testing session took place, including the PANSS and the EmQue. In some of the cases, the iPod was given after the testing session. Two or three days after the start of the ESM, the researchers called the participants to inquire about the progress of filling in the questionnaires, and to encourage the participants to continue with the ESM. When returning the iPod with sufficient data, participants received 25 euros for participation. The study was approved by the Medical Ethics Committee of VU University Medical Center Amsterdam.

### Statistical Analysis

Stata version 14.2 was used for data analysis (StataCorp, 2015). Simple linear regression analyses and chi-squared tests were performed to check for differences in characteristics between the control and PS group. Secondly, multilevel random regression analyses were conducted, in which repeated measures in time were considered as level 1, and participants as level 2. For every hypothesis, three separate outcome measures of mental health were used: positive symptoms, positive affect and negative affect. Age, sex, and education level were included as covariates in all analyses. For the first part of research question 1, the predictor social contact was added as a dichotomous variable (being in contact versus being alone). For the second part, the predictor was close (instead of social) contact (close contact versus less close contact). Furthermore, an interaction between contact and group (PS group versus healthy participants) was added to test for differences between the PS group and healthy participants. If this interaction was significant, analyses were run per group. If the interaction was non-significant, analyses were run excluding the interaction term. For the hypothesis regarding empathy as a moderator (centered composite score of affective and cognitive items), we used the same model but added empathy. Furthermore, we investigated whether there was an interaction between group and empathy, and social/close contact and empathy, for every outcome measure. To correct for multiple testing, we applied the Bonferroni adjustment – i.e., three outcome measures with four analyses each (i.e., social contact, close contact, social contact × empathy, and close contact × empathy) resulting in an alpha level of 0.05/12 = 0.004. See Table 2 for an overview of the models.

**TABLE 2:** Presentation of the models.

Model 1 and 2	Group
	Contact^a^
	Group × Contact^a^
Model 3 and 4	Empathy
	Empathy × Group
	Empathy × Contact^a^

*Model 3 and 4 are model 1 and 2, including empathy as extra factor. Age, sex, and education level were added to all models as covariates. All models were run 3 times: with the outcome measures positive symptoms, positive affect, negative affect.*

*^a^In model 1 and 3 “Contact” refers to the variable social contact. In model 2 and 4 “Contact” refers to closeness of contact.*

## Results

### Participant Characteristics

Demographics and participant characteristics are shown in Table 1. No significant differences between the PS group and healthy control participants were found in terms of sex, age, education level, and country of birth. The PANSS was only assessed in the PS group. Levels of empathy did not differ between the groups.

#### Social Contact and Mental Health

Experience sampling method variables are displayed in Table 3, showing no differences between the control and PS group in number of social contact (*b* = −0.06, *p* = 0.25) and close contact (*b* = 0.07, *p* = 0.22), but the control group filled out significantly more ESM measurements (*p* = 003). The PS group reported more positive symptoms (*b* = 0.77, *p* < 0.001), and more negative affect (*b* = 0.87, *p* < 0.001) than the control group. No differences were found for positive affect (*b* = −0.40, *p* = 0.06).

**TABLE 3:** ESM variables on social contact and mental health compared between the groups.

	Controls (*n* = 28)	PS group (*n* = 29)
Total *n* ESM obs	1,335**	1,018
Mean *n* ESM obs	47.68**	35.10
**Social contact**		
“Yes,” obs, (%)	66.67%	60.32%
**Close contact**		
“Yes,” obs, (%)	65.72%	72.24%
Family, obs (%)	34.73%	33.97%
1 friend, obs (%)	7.47%	11.80%
Roommates, obs (%)	5.41%	5.41%
Friends, obs (%)	14.64%	8.48%
Partner, obs (%)	3.47%*	12.58%
“No,” obs (%)	34.28%	27.76%
Colleagues, obs (%)	11.82%	6.41%
Classmates, obs (%)	15.46%**	5.26%
Stranger, obs (%)	7.00%*	16.09%
**Mental health**		
Positive symptoms, *M* (*SD*)	1.33 (0.35)***	2.09 (0.95)
Positive affect, *M* (*SD*)	4.77 (0.57)^†^	4.31 (0.98)
Negative affect, *M* (*SD*)	1.72 (0.62)***	2.58 (1.14)

*PS group, individuals with psychotic symptoms; ESM, experience sampling method; obs, observations; M, mean; SD, standard deviation. Groups significantly differed in number of ESM completions (p = 0.003). The other comparisons regarding type of contact were not significant after applying Bonferroni correction.*

*^†^p = 0.06.*

*p < 0.05.

**p < 0.01.

***p < 0.001.

### Mental Health

Results are presented in order of the hypotheses, first for social contact, followed by close contact.

### Social Contact

#### Positive Symptoms

The analysis showed no significant interaction effect for social contact and group on positive symptoms, [*b* = −0.0001, 95% CI (−0.09 to 0.09), *p* = 1.00]. Further analyses, excluding the interaction, showed no significant effect for social contact [*b* = 0.04, 95% CI (−0.002 to 0.09), *p* = 0.06], indicating that social contact was not associated with positive symptoms in the total sample.

#### Positive and Negative Affect

There was no significant interaction effect between social contact and group on positive affect [*b* = 0.05, 95% CI (−0.12 to 0.23), *p* = 0.53] nor on negative affect [*b* = 0.01, 95% CI (−0.13 to 0.15), *p* = 0.91]. A main effect was found for social contact on positive affect [*b* = 0.22, 95% CI (0.14 to 0.30), *p* < 0.001], indicating that more social contact was associated with more positive affect, regardless of group. No main effect was found for social contact on negative affect [*b* = −0.06, 95% CI (−0.13 to 0.008), *p* = 0.08].

### Close Contact

#### Positive Symptoms

An interaction effect was found for closeness of contact and group on positive symptoms, [*b* = −0.22, 95% CI (−0.35 to −0.08), *p* = 0.001]. Closeness of contact predicted positive symptoms in the patient group [*b* = −0.24, 95% CI (−0.35 to −0.13), *p* < 0.001], but not in the control group [*b* = −0.03, 95% CI (−0.10 to 0.05), *p* = 0.47], indicating that meeting with close others, as compared to less close others, was associated with lower positive symptoms only in the PS group.

#### Positive and Negative Affect

The analyses revealed no significant interaction of closeness of contact and group on positive affect nor on negative affect [*b* = −0.08, 95% CI (−0.31 to 0.16), *p* = 0.53; *b* = −0.08, 95% CI (−0.28 to 0.11), *p* = 0.40, respectively]. A main effect of closeness of contact on positive affect was found [*b* = 0.20, 95% CI (0.09 to 0.31), *p* < 0.001], but not on negative affect [*b* = −0.08, 95% CI (−0.17 to −0.02), *p* = 0.11], indicating that being with close contacts, as compared to company of less close others, was associated with more positive affect but not with negative affect in the total sample.

### Empathy

#### Social Contact, Empathy, and Positive Symptoms

The analyses showed no interaction effects for group with empathy nor social contact with empathy on positive symptoms (*b’s* ≤ −0.03 and *p’s* ≥ 0.59).

#### Social Contact, Empathy, and Affect

No interaction was found between empathy and social contact on positive affect nor on negative affect (*b’s* < −0.003, *p*’s ≥ 0.83), nor of empathy and group on affect (*b’s* ≥ −0.01 ≤ 0.05 and, *p’s* ≥ 0.50).

#### Closeness of Contact, Empathy, and Positive Symptoms

No significant interaction between empathy and closeness of contact on positive symptoms was found for the total sample [*b* = 0.01, 95% CI (−0.01 to 0.03), *p* = 0.35], nor for empathy and group [*b* = −0.02, 95% CI (−0.14 to 0.10), *p* = 0.79].

#### Closeness of Contact, Empathy, and Affect

**FIGURE 1:**
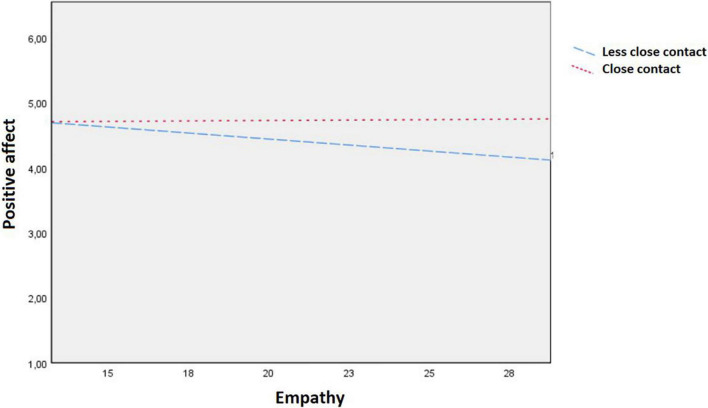
Interaction effect of empathy as measured by the EmQue-CA and closeness of contact on positive affect as measured by the experience sampling method (ESM), for the total sample.

A significant interaction effect was found for empathy and close contact on positive affect for the total sample [*b* = 0.06, 95% CI (0.02 to 0.11), *p* = 0.003, see Figure 1] but not on negative affect for the total sample [*b* = −0.02, 95% CI (−0.05 to 0.02), *p* = 0.41]. There was no interaction between empathy and group on positive affect [*b* = 0.02, 95% CI (−0.11 to 0.16), *p* = 0.73] nor on negative affect [*b* = 0.01, 95% CI (−0.14 to 0.15), *p* = 0.93]. Follow-up analyses were carried out to interpret the interactions as shown in Figure 1 and revealed a non-significant positive association between empathy and positive affect when being in company of close others [*b* = 0.02, 95% CI (−0.05 to 0.09), *p* = 0.58], and a non-significant negative association for being in company of less close others [*b* = −0.02, 95% CI (−0.10 to 0.06), *p* = 0.64].

## Discussion

The current study aimed to gain more insight into the relationship of social contact, closeness of contact, and empathy on mental health in individuals with PS versus controls. The results showed that being in company of others compared to being alone was associated with higher levels of positive affect in the total sample. However, social contact with close others (e.g., friends and partner) was significantly more related to higher positive affect than company of less close others. Furthermore, company of close others was associated with lower positive symptoms in the PS group only. This indicates that the nature of the contact (close versus less close) plays a relevant role, especially for individuals with PS, since it may lower their positive symptoms, although the direction of the effect could not be determined. In addition, the results for the total sample showed an interaction for closeness of contact and empathy on positive affect, suggesting that as levels of empathy increased, positive affect decreased when being in company of less close contacts but not when being in the company of close contacts. However, the results need to be considered with caution as these effects were not significant per type of contact (close versus less close) separately.

The results showed that being in social contact was not related to negative affect but it was related to more positive affect in the total sample. It was also found that close contact compared to less close contact was significantly related to more positive affect for the total sample. Furthermore, contact with close others was related to lower positive symptoms in the patient group only. These findings are in agreement with previous research indicating that contact with close others is associated with a lower symptom severity (Verdoux et al., 2003; Husky et al., 2004; Collip et al., 2011). However, we could not test the direction of the effect. It may therefore also be possible that those who feel better, seek more contact with close others. Further research is needed to investigate the direction of this link. Nonetheless, these findings highlight the importance of the closeness of the contact and seem highly relevant for clinical practice. Therapies could focus more on involving close contacts in the life of the patient. Additionally, the role of empathy was investigated. It was assumed that the PS group would show lower levels of empathy than the control group, but this was not the case. This might be explained by the fact that we used a self-report questionnaire. This may lead to subjective biases and objective measures have been suggested to more accurately measure empathic skills (Chrysikou and Thompson, 2016; Van Donkersgoed et al., 2019). It may also be due to the fact that the PS group consisted of CHR and FEP participants and some studies suggest that empathic deficits are more visible in patients with chronic schizophrenia (Canty et al., 2021). Furthermore, we measured empathy as a trait rather than a state. Future research could incorporate ESM questions measuring empathy to investigate fluctuations of empathy and how it may affect the relationship between contact with others and mental health within one moment. It was hypothesized that individuals with higher levels of empathy benefited more from social contact resulting in a better mental health. Results showed that empathy only moderated the relationship between closeness of contact and positive affect and only for the total sample. The interaction plot suggested that for individuals with higher levels of empathy in combination with being with close others was related to higher positive affect compared to being with less close others. However, the direction of the effect is unclear as we could not investigate causality and more importantly, the effect of empathy on positive affect per type of contact (close versus less close) was non-significant. However, type of contact had a differential effect on positive affect dependent on levels of empathy. More research with larger sample sizes is needed to find a potential effect. Based on our results, empathy does not seem to play a pivotal role in boosting the relationship between contact and mental health.

Our results need to be considered in the light of several limitations. The sample size was relatively small and not all participants filled out every ESM measurement. We found that individuals with PS responded significantly less often than control participants. Although we have no insight in reasons for missing ESM measurements, it might be that with higher symptom severity, individuals with PS were less likely to fill out measurements which in turn may have influenced our results. For future research, we recommend to provide one pop-up question (e.g., multiple choice) for the participant to indicate why he/she did not fill out the measurement. Another point is that the PS group used different types of medication. For example, CHR were on medication for anxiety and depressive symptoms whereas FEP were predominantly taking antipsychotic medication. These factors may have influenced their natural daily behavior and feelings. To avoid these biases, future studies should include more participants, and a more homogeneous group, preferably consisting of unmedicated individuals with PS. As we could not determine the directions of the effects, it would be valuable if future research included longitudinal data to show the temporal sequence, long-term effects and possibly causality. Furthermore, we did not have information regarding the bond of the company the participant was with, although we knew to what extent they felt comfortable in their company. However, it would have been best if participants rated how close they were to the specific person. Additionally, it would be valuable if future research investigated whether treatments involving close contacts are effective in individuals with PS. Furthermore, we were not able to measure perceived social support, but this may be a mediator between social contact and symptom severity as close contacts seem to be related to higher perceived social support (Manago et al., 2012), and social support has been linked to better outcomes in people with mental illnesses (McCorkle et al., 2008). Future research can investigate this link further. It is also interesting to investigate whether negative symptoms decrease when individuals with PS are motivated to engage in social contact with good friends or family. This could be done by providing therapy which involves the close contacts in order to strengthen their relationship and facilitate the contact. Lastly, it would be interesting to divide positive symptoms into manic and paranoid symptoms, as they may differ in terms of corresponding affect.

The current findings lead to several implications for clinical practice. Since the results showed that company of close contacts was associated with better outcomes, therapy should include ways to utilize these beneficial contexts either directly by involving close friends/family in therapy or indirectly by guiding the patient in seeking contact with close others. In the United Kingdom, family therapy is already a recommended psychological treatment for individuals with psychosis [National Institute for Health and Care Excellence (NICE), 2015].

In summary, we can conclude that social contact, especially with close others, is linked to a higher positive affect for both individuals with PS and healthy controls. Additionally, in individuals with PS, only close contacts were related to less positive symptoms. This seems an important finding for clinical practice as therapy could focus on this aspect, though we did not investigate the causality or long-term effects of this association. Levels of empathy might also play a role in terms of positive affect, but this should be further investigated in order to draw firm conclusions.

## Data Availability Statement

The data analyzed in this study can be shared upon reasonable request. Requests to access these datasets should be directed to IL-J and LJK, i.l.j.jansen@vu.nl and l.j.g.krijnen@uu.nl.

## Ethics Statement

The studies involving human participants were reviewed and approved by the Medical Ethics Committee of the VU University Medical Center Amsterdam. Written informed consent to participate in this study was provided by the participant, and if under the age of 18, also by the participants’ parent or guardian.

## Author Contributions

LJK: conceptualization, methodology, formal analysis, and writing – original draft. IL-J: conceptualization, methodology, formal analysis, and writing – review and editing. A-KF: writing – review and editing. LK: conceptualization, writing – review and editing, and funding acquisition. All authors contributed to the article and approved the submitted version.

## Conflict of Interest

The authors declare that the research was conducted in the absence of any commercial or financial relationships that could be construed as a potential conflict of interest.

## Publisher’s Note

All claims expressed in this article are solely those of the authors and do not necessarily represent those of their affiliated organizations, or those of the publisher, the editors and the reviewers. Any product that may be evaluated in this article, or claim that may be made by its manufacturer, is not guaranteed or endorsed by the publisher.

## Appendix A. ESM Items Used in the Current Study

“I feel comfortable in this company” “With whom am I now?” Alone/Alone with pet/colleague’s/classmates/friends/1 friend/partner/family/roommates/stranger

*Positive symptoms*
“I sense that others do not like me” “I sense that others intend to harm me” “I am feeling suspicious” “I feel surreal” “My thoughts won’t let me go” “My thoughts are influenced by others” “I hear voices” “I see appearances”

*Positive affect*
“I feel lively” “I feel relaxed” “I feel content” “I like myself”

*Negative affect*
“I feel insecure” “I feel anxious” “I feel irritated” “I feel down”

## Appendix B. Participants’ Answers Regarding How Comfortable They Felt With Their Company

**Table:**

	Controls (*n* = 28)		PS group (*n* = 29)	
I feel comfortable in this company	Close contact	Not a close contact	Close contact	Not a close contact
No (scores 1–2)	9 (1.50%)	6 (2.1%)	5 (1.10%)	16 (10.40%)
Medium (scores 3, 4, 5)	47 (7.85%)	86 (29.66%)	168 (35.97%)	82 (53.25%)
Yes (scores 6–7)	543 (90.65%)	198 (68.28%)	294 (62.96%)	56 (36.36%)
Total obs, *n*	599	290	467	154

*PS, individuals with psychotic symptoms; obs, number of ESM observations. Participants felt significantly more comfortable with company of close contacts (p < 0.001).*
